# Pediatric Telehealth Expansion in Response to COVID-19

**DOI:** 10.3389/fped.2021.642089

**Published:** 2021-09-17

**Authors:** Stormee Williams, Kristina Hill, Luyu Xie, M. Sunil Mathew, Ashley Ofori, Tamara Perry, Danielle Wesley, Sarah E. Messiah

**Affiliations:** ^1^Children's Health System of Texas, Dallas, TX, United States; ^2^School of Public Health, University of Texas Health Science Center at Houston, Dallas, TX, United States; ^3^Center for Pediatric Population Health, Children's Health System of Texas and University of Texas Health Science Center, Dallas, TX, United States

**Keywords:** telehealth, pediatric, expansion, COVID-19, healthcare, children, adolescents

## Abstract

**Introduction:** Telehealth utilization has been steadily increasing for the past two decades and has been recognized for its ability to access rural and underserved populations. The advent of COVID-19 in March 2020 limited the feasibility of in-person healthcare visits which in turn increased telehealth demand and use. However, the long-term impacts of COVID-19 on the telehealth sector of the healthcare industry, and particularly on pediatric healthcare volume demand and subsequent expansion, are yet to be determined.

**Objective and Methods:** To understand the impact of COVID-19 on telehealth utilization, volume demand, and expansion in one large pediatric healthcare system serving greater Dallas-Fort Worth, Texas, data on telehealth clinic visits by month, pre-COVID and post/current-COVID were compared. A quasi-experimental pretest-posttest design analysis compared telehealth visit counts from 54 ambulatory pediatric health specialties. Pre-post new patient counts were also analyzed via chi square.

**Results:** Total telehealth visit counts significantly increased between March–October 2019 (2,033 visits) compared to March-October 2020 (54,276 visits). Mean monthly telehealth visits increased by 6,530 visits, or 2,569.75% over the same time period (*p* < 0.0001). In October 2020, total telehealth visits were still 1,194.78% above 2019 levels (345 visits in 2019 vs. 4467 visits in 2020).

**Discussion:** Results here show a substantial volume increase in telehealth-delivered pediatric healthcare and resource utilization as a response to COVID-19. This provides a template for permanent adoption of pediatric telehealth delivery post pandemic. Further investigation is needed to determine impacts upon resource allocation, processes, and general models and standard of care to assist facilities and programs to better address the needs of the pediatric populations they serve in the post-COVID era.

## Introduction

Telehealth is the use of telecommunication technology to provide long distance health care services. In the past decade, there has been a dramatic expansion of telehealth services in the United States (1). Generally, telehealth services are delivered using technological tools such as live video teleconferencing, store-and-forward technology, remote patient monitoring, telephone, mobile health applications, text, and email (2, 3). These tools enable providers to deliver clinical services and treatment to patients remotely in an efficient manner.

Telehealth has proven to be effective in managing acute infections, rapid pediatric triage in the emergency department, and providing positive mental health, primary care, cardiology, and dermatology outcomes (4). According to Polinski et al. (4), many patients are satisfied with the quality of care they receive through telehealth and find that their quality of care is comparable to that of traditional in-person care. Many patients prefer the convenience of telehealth services to those of traditional hospital care (4). Consequently, telehealth utilization by hospital systems in the United States has increased from 35% to 76% from 2010 to 2017 (5). Additionally, telehealth insurance claims increased by 53% from 2016 to 2017 (5). Studies indicate that telehealth care is cost-effective, especially when used for psychiatric care, radiology, and home healthcare services (2). It is also effective in reducing cost of travel and time for medical care, hospital utilization, improved patient compliance, satisfaction, and chronic disease management (6).

The efficacy and effectiveness of telehealth has consequently facilitated the implementation of telehealth programs in many pediatric hospitals, child care centers, and schools (7, 8). The incorporation of telemedicine in pediatric settings and school-based programs has been shown to reduce absenteeism, improve patient satisfaction, provide cost savings, reduce emergency department visits, and offer time savings for parents (9). Currently, medical subspecialties such as pediatric dermatology, emergency medicine, intensive care, neonatology, cardiology, surgery, and psychiatry are commonly known to utilize telemedicine (7).

Despite the many advantages and the growing use of telehealth in pediatric health, several studies have suggested that pediatric healthcare volume demand and expansion have been stifled by restrictive laws and regulations, payment structures, and reimbursement issues (10–12). Only 15% of pediatricians in a 2016 study reported having used telehealth (12). The most commonly reported barriers to telehealth adoption were insufficient payments and billing issues.

With the advent and spread of the novel coronavirus disease 2019 (COVID-19) in March 2020—which has limited the feasibility of in-person healthcare visits across the United States—telehealth demand and use has become increasingly important to meeting pediatric health care needs nationwide. School closures have given telehealth particular relevance for the pediatric population. Indeed, in a guidance issued on March 18, 2020, by the American Academy of Pediatrics, pediatricians were directed to increase telehealth care services to meet health care demands. This recommendation facilitated coverage expansion and relaxation of telehealth regulations in many states, and the expansion of Medicaid programs and other insurance payers which previously were barriers to telehealth expansion for pediatricians (11, 12). Thus, this guidance has opened opportunities for a potential rapid surge in telehealth utilization in pediatric health care delivery.

Though many recent studies and reports have alluded to the reduction in barriers to telehealth, and its general increased usage due to COVID-19 restrictions, an estimate of telehealth volume demand pre-post COVID-19 for pediatric health delivery has not been widely reported. The goal of this study is to assess pediatric healthcare volume demand and subsequent expansion before and after the advent of COVID-19 within one large pediatric healthcare system serving greater Dallas-Fort Worth, Texas. Findings from this study could provide evidence and direction for extent of adoption of pediatric telehealth delivery post the COVID-19 pandemic.

## Methods

### Study Design

In March 2020, as COVID-19 spread quickly across the world, leaders at Children's Health System of Texas acknowledged the benefit telehealth could bring to the thousands of children and families that are served locally and regionally. It was decided to expand telehealth offerings beyond the previous 15 service areas to include most, if not all, of the 70 ambulatory service lines within the health system. To understand the implications and impact of the COVID-19 pandemic on telehealth utilization within a major pediatric health care system, we performed a quasi-experimental pretest-posttest design analysis in which the number of telehealth visits and the number of pediatric specialties performing these visits were reviewed and compared.

### Procedure

To expand telehealth services rapidly, we had to quickly make assessments of existing resources including technology needs, staffing—both clinical and non-clinical—and training materials, and align those with the policy/credentialing, regulatory, and management needs of the system. Prior to COVID-19, there were 15 service lines already able to use telemedicine. During the pandemic, that number extended to 54 individual service lines (Table 1). A systematic approach was utilized to ensure that the individual physicians and staff received the appropriate training and credentialing they needed to use the technology to provide telemedicine care. This approach utilized teams of hospital managers and clinical advisors that worked to prioritize which service lines would receive the training and credentialing, and in what order. Staff in multiple departments worked around the clock to compress the work activities necessary to get physicians and frontline staff ready to use telemedicine.

**Table 1:** Ambulatory telemedicine groups/clinics, Children's Health System of Texas.

**Group or Clinic**	**Group or Clinic**
Allergy and Immunology	Nephrology
Andrews Institute Orthopedics and Sports Medicine	Neuroimmunology
Clinic for At Risk Children	Neuropsychology
Clinic for At Risk Children—Psychology	Nutrition
Audiology	Our Children's House Therapy Services
Autism and Developmental Disabilities	Orthopedic Surgery
Autism and Developmental Disabilities—Neurology	Orthopedics
Autism and Developmental Disabilities—Psychology	Pediatric (General) Surgery
Cardiology	Pediatric Cardio Surgery
Cardiology—Pediatric Cardiology Associates of Houston	Pediatric Neurology
Cardiology—Pediatric Health Specialists	Pediatric Neurosurgery
Cityville Neurology	Pediatric Urology
Complex Care	Plano Allergy/Ear, Nose and Throat
Dermatology	Plastic Surgery
Developmental and Behavioral Pediatrics	Pre-Operative
Dallas Physicians Medical Services for Children	Psychiatry
Endocrinology	Psychology
Ear, Nose and Throat	Physical/Occupational/Speech Therapy
Emergency Room Center	Pulmonology
The FETAL (Fetal Evaluation and Treatment Alliance) Center	Rheumatology
General Pediatrics	Sleep Clinic
Genetics	Solid Organ Transplant
Gastrointestinal	Speech Language Pathology—Cityville
Gynecology	Surgery Specialty Center
Hematology and Oncology	Foster Care Clinic
Infectious Diseases	Thrive Post-NICU Clinic
Medical District Primary Care	Virtual Health

Working along with clinical leadership in the ambulatory clinics, a prioritized list was drafted for the development of each virtual clinic. Prioritization was determined by clinical care severity levels. Using this method, cardiology, neurology, and solid organ transplant were among the first clinics implemented. During this rapid deployment, there were no exclusion criteria for telehealth visits implemented as federal and state mandates were in place to open visits to both audio and video-based visits. In addition, all telehealth visits could be billed under the emergency status which broadened the visit type allowance. Generally, visits would need to be via video for billing and reimbursement to occur in the ambulatory clinical setting.

During the expansion and rollout of the telehealth clinics, monitoring was put in place to track metrics such as number of telehealth visits, visit type, length of video/audio consult, and connection issues (dropped calls, platform disconnects, inability to use video, etc.). Daily and weekly meetings were convened to discuss recent trends and to implement any necessary changes quickly as the need arose. These data were analyzed in this study to provide insight on the impact of COVID-19 on telehealth utilization.

### Statistical Analysis

Descriptive analysis was performed for patients' characteristics including age, sex, race/ethnicities (non-Hispanic white [NHW], non-Hispanic black [NHB], Hispanic, and other), preferred languages (English, Spanish, and other), insurance (government, commercial, and self-pay) and major diagnostic categories.

Pre-COVID vs. post-COVID visit counts were analyzed via chi square using Stata (copyright 2020, StataCorp LLC). Telehealth visit counts were grouped bimonthly (January-February, March-April, etc.). Bimonthly counts were compared from January 2019 through October 2020 (the final month with data available at time of manuscript writing). The primary results were taken from the comparison of the telehealth visit counts at the same time of year between 2019 and 2020, in order to account for possible fluctuations independent of the influence of the pandemic in healthcare and/or telehealth usage due to time of year, which might skew results. March 2020 marked the advent of COVID-19 in the areas served by the Children's Health System of Texas, and the period of marked increase for pediatric telehealth visits; therefore, focus was given to March-October of 2020 as compared with the same period in 2019, to show the full impact of the telehealth expansion pre- and post-COVID onset.

## Results

Table 2 shows the telehealth patients' sociodemographic information and major diagnostic categories where available. There were 1,779 and 43,997 telehealth visits between January and October of 2019 and 2020, respectively (total *n* = 45,772) for which demographic data were collected. Mean age was 11.8 years (standard deviation 9.3), 50.7% of the population were male, and the majority were non-Hispanic White (32.8%) or Hispanic (31.8%). Most patients preferred to speak English (79.3%), lived in Texas (98.5%), and were covered by either government insurance (54.8%) or commercial insurance (44.5%). In 2019, all patients had urgent care telehealth visits, so the diagnostic information were not available. In 2020, from the onset of the pandemic onward, the primary diagnoses were mental or neurodevelopmental disorders (22.2%), followed by endocrine, nutritional, and metabolic disorders (15.4%), digestive disease (9.6%), and respiratory disease (9.3%).

**Table 2:** Characteristics of patients based on telehealth visits, Jan–Oct 2019 and 2020.

	**2019 (*n* = 1,775)**	**2020 (*n* = 43,997)**	**Total (*n* = 45,772)^**a**^**
**Age at visit, years, mean (SD)**	31.2 (11.7)	11.0 (8.1)	11.8 (9.3)
**Sex**, ***n*****(%)**			
Female	1,353 (76.2)	21,200 (48.2)	22,553 (49.3)
Male	422 (23.8)	22,790 (51.8)	23,212 (50.7)
Unknown	0 (0)	7 (0.02)	7 (0.02)
**Race/ethnicity**, ***n*****(%)**			
Non-Hispanic white	158 (8.9)	14,859 (33.8)	15,017 (32.8)
Non-Hispanic black	31 (1.8)	8,703 (19.8)	8,734 (19.1)
Hispanic	83 (4.7)	14,456 (32.9)	14,539 (31.8)
Other/unknown	1,503 (84.7)	5,979 (13.6)	7,482 (16.4)
**Parent preferred language**, ***n*****(%)**			
English	333 (18.8)	35,977 (81.8)	36,310 (79.3)
Spanish	5 (0.3)	5,543 (12.6)	5,548 (12.1)
Other	1,437 (81.0)	2,477 (5.6)	3,914 (8.6)
**State**, ***n*****(%)**			
Texas	1,761 (99.2)	43,341 (98.5)	45,102 (98.5)
Other	14 (0.8)	656 (1.5)	670 (1.5)
**Insurance**, ***n*****(%)**			
Government	1 (0)	25,066 (56.9)	25,067 (54.8)
Commercial	1,769 (99.7)	18,584 (42.2)	20,353 (44.5)
Self-pay/unknown	5 (0.3)	347 (0.8)	352 (0.8)
**Primary diagnosis**, ***n*****(%)**			
Infectious disease	N/A^b^	322 (0.7)	322 (0.7)
Neoplasms		339 (0.8)	339 (0.8)
Diseases of the blood and blood-forming organs		695 (1.6)	695 (1.5)
Endocrine, nutritional, and metabolic disease		6,776 (15.4)	6,776 (14.8)
Mental and neurodevelopmental disorders		9,782 (22.2)	9,782 (21.4)
Disease of eye and ear		1,173 (2.7)	1,173 (2.6)
Circulatory system disease		1,049 (2.4)	1,049 (2.3)
Respiratory system disease		4,070 (9.3)	4,070 (8.9)
Digestive system disease		4,415 (9.6)	4,415(9.6)

*^a^The total visits in this table do not include telehealth urgent care patient visits from 2019 and 2020.*

*^b^The primary diagnosis for telehealth visits in 2019 is not available in the electronic health record*.

**Figure 1:**
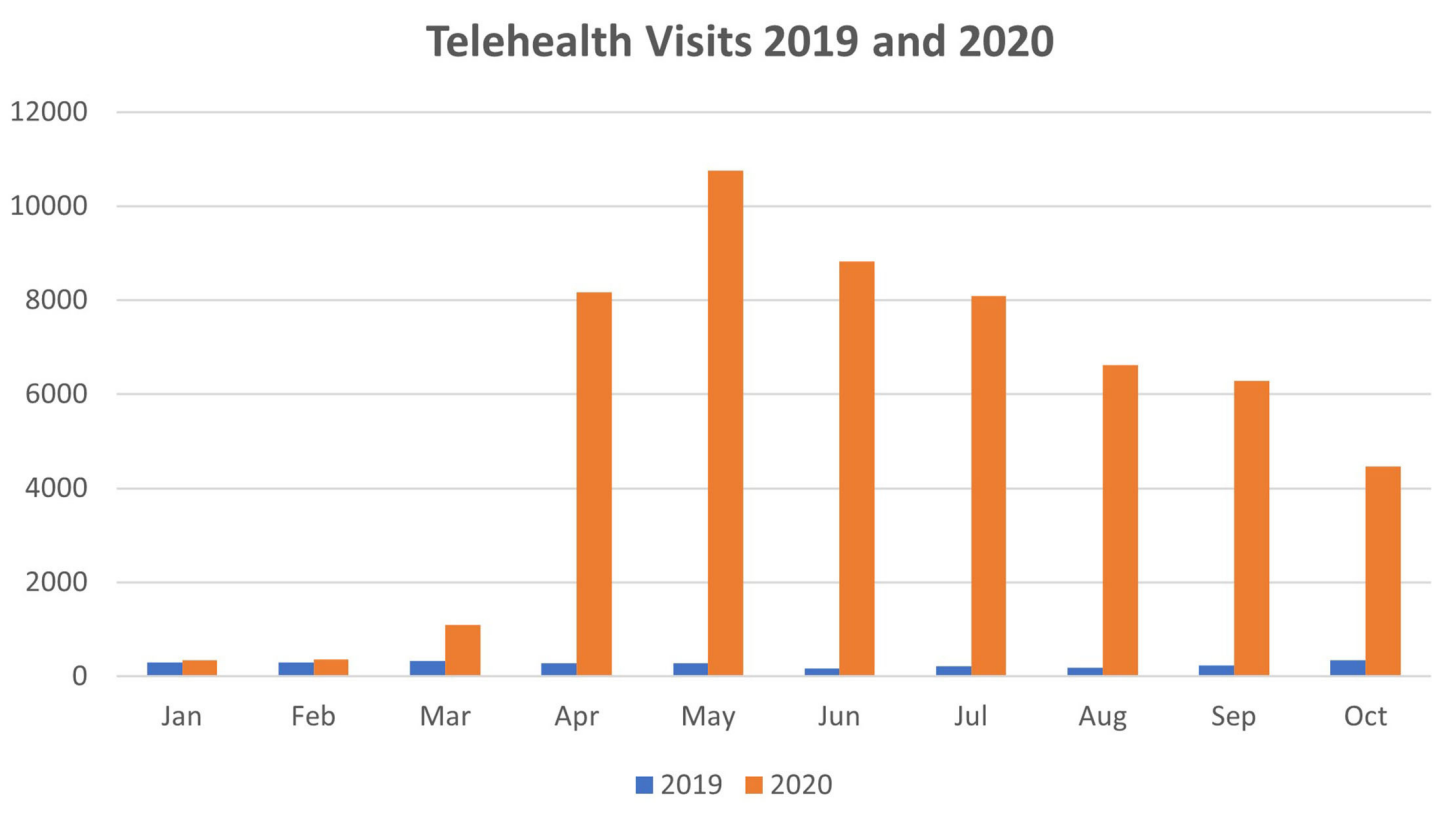
Telehealth visits by month, 2019 and 2020.

Total telehealth visit counts significantly increased in the eight-month periods between March-October 2019 (2,033 total telehealth visits) compared to March-October 2020 (54,276 total telehealth visits). Mean monthly telehealth visits increased by 6,530 visits, or 2,569.75% when comparing the same two time periods (*p* < 0.0001). In October 2020, total telehealth visits were still 1,194.78% above 2019 levels (345 visits in 2019 vs. 4,467 visits in 2019). Telehealth visit counts for January-October of both years are shown in Table 3 and Figure 1.

**Table 3:** Telehealth visit counts in 2019 vs. 2020.

**Month**	**2019**	**2020**	**% change**
Jan	298	343	15.10
Feb	301	369	22.59
Mar	322	1,094	239.75
Apr	283	8,163	2,784.45
May	275	10,751	3,809.45
Jun	168	8,823	5,151.79
Jul	217	8,086	3,626.27
Aug	188	6,614	3,418.09
Sep	235	6,278	2,571.49
Oct	345	4,467	1,194.78

Telehealth visit counts in 2019 were also compared in two-month groupings via Chi-square against the same 2 months in 2020. Differences were not significant between January/February 2019 and January/February 2020 (both periods pre-COVID; Chi-square value 0.3231, *p*-value = 0.570). However, differences were stark from March/April 2019 compared to March/April 2020 (Chi-square value 791.7409, *p*-value < 0.0001). This trend continued for all 2-month groups excepting July/August 2019 vs. July/August 2020 (Chi-square value 0.3240, *p*-value 0.569). Full results of these comparisons are shown in Table 4.

**Table 4:** Telehealth visit counts in 2019 and 2020^*^.

**Month**	**Jan–Feb 2020**		**Mar–Apr 2020**		**May–Jun 2020**		**Jul–Aug 2020**		**Sep–Oct 2020**	
	**Chi-square statistics**	***p*** **-values**	**Chi-square statistics**	***p*** **-values**	**Chi-square statistics**	***p*** **-values**	**Chi-square statistics**	***p*** **-values**	**Chi-square statistics**	***p-*** **values**
Jan–Feb 2019	0.3231	0.570	667.3949	0.000	6.2841	0.012	6.4221	0.011	17.5348	0.000
Mar–Apr 2019	3.3355	0.068	791.7409	0.000	0.6863	0.407	0.7468	0.388	6.3738	0.012
May–Jun 2019	21.2175	0.000	880.9820	0.000	8.9556	0.003	8.6926	0.003	2.3354	0.126
Jul–Aug 2019	3.0179	0.082	577.0549	0.000	0.2898	0.590	0.3240	0.569	3.7703	0.052
Sep–Oct 2019	7.5797	0.006	384.7218	0.000	47.1565	0.000	47.2342	0.000	72.2360	0.000

* *Chi-square statistics and p-values for the difference between bimonthly visit counts in 2019 and 2020*.

## Discussion

Results here show a substantial volume increase in telehealth-delivered pediatric healthcare and resource utilization as a response to COVID-19. The differences are not explained purely by year upon year increases in telehealth usage, as seen by the lack of statistical difference between January/February 2019 and January/February 2020 periods (both unimpacted by COVID-19) as opposed to the distinctions seen between the remainder of 2019 and the corresponding months in 2020. Trends in utilization of telehealth in pediatric care pre-COVID, already on the rise, have been accelerated dramatically by the pandemic (13, 14). Healthcare providers may benefit from a template for permanent adoption of pediatric telehealth delivery post pandemic.

Children's Health System of Texas has a long history of providing telehealth services. Beginning in 2013 with its tele-neonatology and tele-emergency programs that focused on providing doctor-to-doctor e-consults to referring hospitals, and a robust school-based telehealth program that has served over 15,000 patients in North Texas since its inception, the focus of the telehealth offerings were aimed at strategic priorities—reducing unnecessary patient transfers and emergency department visits. In 2015, telehealth services expanded to include direct-to-consumer telehealth visits, first to employees then for community pediatric and adult patients within the state of Texas, providing both urgent care and behavioral health consults. It was during this time that telemedicine services were offered to various subspecialties within the health system, while facing the ever-evolving reimbursement and regulatory challenges that were common across the country. With these challenges, the uptake of telemedicine throughout the health system was less than ideal.

The COVID-19 pandemic increased patient demand for telehealth services dramatically, as well as our health system's need to shift our paradigm in order to help keep our patients and staff safe. Swift rollout of the telehealth service lines was crucial in this period. Typically, the training process for telehealth rollout takes 2–3 weeks to complete per service line. In the face of the pandemic, this timeline was shortened to <1 week for each service line. One of the changes in procedure which allowed this rapid ramp-up was to train and credential the providers by department, as opposed to individual physicians on a first-come, first-served basis. Additionally, virtual training courses were developed to shorten the training cycle and could be completed by the providers around the clock. Training occurred separately for each provider and included a virtual mock visit with a qualified staff member. As many as 2000 providers were trained in total during this period across the 54 clinical departments involved.

The need and demand for telehealth in our health system mimics that of myriad health centers throughout the country which have had to adapt to the ever-changing and uncertain environment brought about by the pandemic. The long-term impact on telehealth utilization across the country is yet to be seen, but given the shifts in infrastructure, process, and resourcing that have occurred, it is likely that our system, and many others nationwide, will be utilizing telehealth at much higher rates than pre-pandemic in the coming months and years.

Further investigation is needed to determine long-term impacts upon resource allocation, processes, and general models and standard of care that COVID-19 has wrought in pediatric healthcare in the relatively short period since March 2020, as well as upon patient outcomes and management of pediatric care, as patients and practitioners alike adjust to the positive and negative aspects of telehealth as opposed to in-clinic visits. Better understanding of these impacts and models of rapid change such as those described above may assist facilities and programs nationwide to better address the needs of the pediatric populations they serve through telehealth in the coming months, and beyond in the post-COVID era.

### Study Limitations and Strengths

Analysis was generated from a single pediatric healthcare system and therefore may not be generalizable to all others. However, Children's Health System of Texas covers a very populous area, with pediatric population over 60% Medicaid and primarily ethnic minorities (non-Hispanic black and Hispanic patients combined). Other pediatric healthcare systems with similarly socioeconomically and racially diverse makeup may benefit from observation of the model described here. Detailed information on socioeconomic status of patients were not available for this analysis, nor were information on other clinical characteristics of the patient population. The results of the study were captured in the short term over just a few months. Long-term ramifications of this drastic increase in pediatric telehealth services will be observed over the coming months and years in our system as well as others. Needed adaptations to the model described here will undoubtedly surface over that period in the changing environment.

Although the primary diagnosis information from 2019 are not available to compare by service line against that from 2020 for the purposes of this study, it is nonetheless noteworthy that the telehealth program instituted across all service lines used video only, without peripheral devices that can aid in diagnostic capabilities. This limits the types of diagnoses that can be adequately assessed and treated remotely. For example, mental and neurological diseases make up a large proportion of all visits due to the feasibility of diagnosing over video and discussing through counseling via telehealth, as opposed to diseases in in which a hands-on physical exam is needed. As healthcare systems, including our own, assess the long-term impacts of COVID-19 on their telehealth programs, the technologies utilized may adapt to better address these current functional limitations and may lead to areas of future study.

A result of the study that may become clearer when summer 2021 data are available to compare against is related to the difference between July/August 2019 and July/August 2020, which lacks statistical significance as per Table 4. Although the reason for the lapse during these months is unknown, potential contributing factors include seasonal downtick in telemedicine usage generally and downtick in COVID-19 transmission during the warmer months of summer 2020.

Future potential areas of study outside the scope and reach of this project include analyses of the utilization and impact on condition control between separate specialty clinics employing telehealth; vaccination rates of telehealth patients vs. those among in-clinic patients; and relevant prescription information related to these telehealth visits as compared with in-clinic visits. In addition, long-term diagnosis and demographic patterns tracked as the telehealth program continues to expand may be illustrative of areas for further study within the patient population addressed here.

## Conclusions

Results here show a substantial volume increase in telehealth-delivered pediatric healthcare and resource utilization as a response to COVID-19 in one of the largest pediatric health care systems in the United States. This provides a template for permanent adoption of pediatric telehealth delivery post pandemic. Further investigation is needed to determine impacts upon resource allocation, processes, and general models and standard of care to assist facilities and programs to better address the needs of the pediatric populations they serve in the post-COVID era. New models of care, including expanded telehealth, arising as the result of the pandemic should be evaluated for effectiveness, return on investment, and impact on quality, in order to determine standards for the new paradigm that best protect the interests of patients and other stakeholders involved in pediatric healthcare.

## Data Availability Statement

The raw data supporting the conclusions of this article will be made available by the authors, without undue reservation.

## Author Contributions

SW and SM conceptualized the study. KH and LX conducted the analysis with oversight from SM. MSM and AO oversaw all data management and IRB aspects of the study. TP and DW oversaw all legal aspects of the project. All authors contributed intellectual property, writing to the final manuscript, and approved submission of the final manuscript.

## Conflict of Interest

The authors declare that the research was conducted in the absence of any commercial or financial relationships that could be construed as a potential conflict of interest.

## Publisher's Note

All claims expressed in this article are solely those of the authors and do not necessarily represent those of their affiliated organizations, or those of the publisher, the editors and the reviewers. Any product that may be evaluated in this article, or claim that may be made by its manufacturer, is not guaranteed or endorsed by the publisher.
